# Enucleation Due to Ocular Abscess in a Captive Chimpanzee (*Pan troglodytes*): A Case Report from the Republic of Congo

**DOI:** 10.3390/vetsci12090805

**Published:** 2025-08-25

**Authors:** Manuel Fuertes-Recuero, José L. López-Hernández, Alejandra Ramírez-Lago, Luna Gutiérrez-Cepeda, Juan A. De Pablo-Moreno, Pablo Morón-Elorza, Luis Revuelta, Rebeca Atencia

**Affiliations:** 1 Department of Physiology, College of Veterinary Medicine, Complutense University of Madrid, Avda. Puerta de Hierro s/n, 28040 Madrid, Spain; manufuer@ucm.es (M.F.-R.); lrevuelt@ucm.es (L.R.); 2 Veterinary Teaching Hospital, Complutense University of Madrid, Avda. Puerta de Hierro s/n, 28040 Madrid, Spain; joslop02@ucm.es; 3 Jane Goodall Institute Congo, Pointe-Noire, Congo; rorilago92@gmail.com (A.R.-L.); rebecatencia@hotmail.com (R.A.); 4 Department of Animal Medicine and Surgery, College of Veterinary Medicine, Complutense University of Madrid, Avda. Puerta de Hierro s/n, 28040 Madrid, Spain; lunaguti@ucm.es; 5 Department of Genetics, Physiology and Microbiology, School of Biological Sciences, Complutense University of Madrid, 28040 Madrid, Spain; 6 Department of Veterinary Sciences, School of Biomedical and Health Sciences, Universidad Europea de Madrid, 28670 Villaviciosa de Odón, Spain; 7 Department of Pharmacology and Toxicology, Complutense University of Madrid, Avda. Puerta de Hierro s/n, 28040 Madrid, Spain; pmoron01@ucm.es; 8 Fundación Oceanográfic, Oceanogràfic of Valencia, C/d’Eduardo Primo Yúfera, 1, Quatre Carreres, 46013 Valencia, Spain

**Keywords:** primate, great ape, surgery, anesthesia, orbital abscess, sanctuary medicine, illegal wildlife trade

## Abstract

Chimpanzees (*Pan troglodytes*) rescued from the illegal wildlife trade often suffer from chronic, traumatic injuries that require specialized and prolonged medical treatment in wildlife rehabilitation centers. We present the case report of a two-year-old male chimpanzee admitted at the Tchimpounga Chimpanzee Rehabilitation Center in the Republic of Congo with a chronic periorbital abscess, likely caused by a machete wound sustained during the poaching of his mother. Despite receiving extended antimicrobial therapy, his condition was never fully controlled and progressed to a chronic orbital infection, causing him discomfort and producing chronic purulent discharge. Enucleation was performed under general anesthesia using ketamine and medetomidine, with surgical approach adapted to the distinctive orbital anatomy of chimpanzees. During the procedure, ligation of the optic nerve and ophthalmic vessels was required due to the confined orbital apex and extensive vascularization, ensuring adequate haemostasias and procedural safety. The chimpanzee made an uneventful postoperative recovery, resuming normal feeding and social behavior within 48 h, with complete wound healing occurring within two weeks. This case report highlights the importance of prompt surgical intervention when conservative medical management fails to resolve refractory ocular infections in chimpanzees. It also emphasizes the importance of specific anesthetic protocols, refined surgical techniques and tailored postoperative care in wildlife rehabilitation centers. Documenting and sharing detailed case reports such as this contributes to the limited veterinary literature on great ape surgery and supports evidence-based clinical decision-making to improve the welfare and treatment outcomes of rescued chimpanzees.

## Simple Summary

Chimpanzees rescued from illegal wildlife trafficking often arrive severely injured to rehabilitation centers. This report describes the case of a young chimpanzee in the Republic of Congo who developed a painful ocular infection following a traumatic injury, most likely sustained when his mother was poached. Despite receiving extensive medical treatment, the infection was unsuccessfully controlled and progressively worsened. Veterinarians performed surgery to remove the affected eye, using techniques adapted to the specific anatomy of chimpanzees. The chimpanzee made a full recovery within two weeks, resuming vital activities such as eating and socializing within two days. This case shows that, when medical route treatment fails, surgical approach can be safely and successfully carried out in wildlife animal rescue sanctuaries. It also highlights the need for specialized veterinary care and proper monitoring of wild animals. Sharing clinical cases as such mentioned one improves the further care of endangered species and wildlife protection.

## 1. Introduction

Chimpanzees (*Pan troglodytes*) are large-bodied hominids belonging to the family Hominidae [1]. Their phylogenetic proximity with humans, cognitive abilities and complex social behaviors make them invaluable in ethological, conservation and biomedical research [2,3,4]. However, chimpanzees are classified as endangered by the IUCN [5] and face a multitude of anthropogenic threats across their range. The illegal trade of live chimpanzees for the exotic pet market and commercial entertainment, the hunting of chimpanzees for their meat (bushmeat), and the use of their body parts in traditional medicine have all contributed significantly to the decline of wild populations [6,7]. Consumption of chimpanzee meat is often driven by cultural practices and is exacerbated during periods of civil instability and food insecurity [6]. This represents a major conservation and public health concern as transmission of zoonotic diseases is increased while populations are further reduced [8]. Chimpanzees are vulnerable to fatal outcomes beyond direct persecution such as snaring, illegal trafficking, bushmeat hunting, vehicle collisions and retaliatory killings related to crop raiding [6]. These ongoing pressures highlight the vital role of sanctuaries and zoological institutions in providing care for rescued chimpanzees, while also contributing to broader law enforcement efforts and conservation strategies [9,10].

Although captive management is essential for the rescue, rehabilitation, and long-term care of displaced individuals, it presents unique and complex challenges to chimpanzee welfare. Due to their physiological, behavioral, and emotional complexity, they require highly specialized, multidisciplinary care [11,12]. Many individuals arriving at wildlife rescue centers are already in a compromised state, presenting with traumatic injuries, infectious diseases, or chronic disorders acquired during the illegal trade, transport, or prior conditions of captivity. Infant and juvenile chimpanzees are often violently separated from their mothers during poaching events, resulting in physical trauma including fractures, lacerations, or soft tissue injuries caused by machetes or knives, as well as profound psychological distress [6,7], which predispose individuals to both acute and pathological changes. Traumatic wounds, often sustained prior to arrival at wildlife rescue centers, are prevalent among rehabilitated chimpanzees. These lesions may progress to chronic infections or abscesses if inadequately treated or when located in delicate anatomical regions. Ocular disease in non-human primates is frequently underreported, despite being clinically significant given the role of vision in social interaction, environmental awareness and psychological well-being [13,14,15,16,17,18,19]. Among ocular conditions, orbital cellulitis and abscess formation pose particular therapeutic challenges due to the limited access to advanced imaging modalities and specialized surgical equipment in remote or resource-limited rehabilitation settings [11].

Reports of medical and surgical intervention in captive chimpanzees remain limited [11,12,20,21,22], particularly concerning orbital pathologies [16]. Nevertheless, several case reports and reviews have outlined anesthetic protocols tailored for great apes, emphasizing the importance of tailored drug dosages, fluid therapy, and comprehensive perioperative monitoring strategies [23]. Anesthetic induction protocols combining ketamine, medetomidine or xylazine are widely reported as safe and effective in both rehabilitation centers and laboratory settings [24,25,26,27,28].

Chimpanzees have a unique orbital anatomy [29,30,31]. The scarcity of published surgical descriptions in non-human primates has constrained the adaptation of human enucleation techniques available in these species. The chimpanzee orbit is deep, conical, and fully enclosed by bone, composed primarily of the frontal, zygomatic, sphenoid, maxillary, lacrimal, and ethmoid bones. This complex bony architecture creates a restricted surgical field with dense fascial and muscular attachments, which requires a meticulous and controlled dissection.

This case report provides a novel and comprehensive description of the initial clinical presentation, diagnostic approach, and successful medical and surgical management of a chronic periorbital abscess in a young (2-year-old) male chimpanzee. The report provides practical guidance for veterinary teams working in wildlife rehabilitation centers, emphasizing the importance of adapting protocols to the unique anatomical, physiological, and behavioral characteristics of great apes.

## 2. Case Presentation

A 2-year-old, 3.8 kg male orphaned chimpanzee (*Pan troglodytes*, a primate belonging to the great ape family *Hominidae*) was transferred to the Tchimpounga Chimpanzee Rehabilitation Center in the Republic of Congo. The individual was rescued from the Cabinda region, Angola, originating from the jungle where his mother had been found deceased. Upon arrival, a comprehensive routine health screening was conducted, and preventive medical protocols were promptly implemented. The chimpanzee was dewormed using fenbendazole (50 mg/kg orally for three days, repeated quarterly) and ivermectin (200 µg/kg subcutaneously biannually). He was also vaccinated against tetanus, hepatitis B, rabies, and poliovirus.

**Figure 1:**
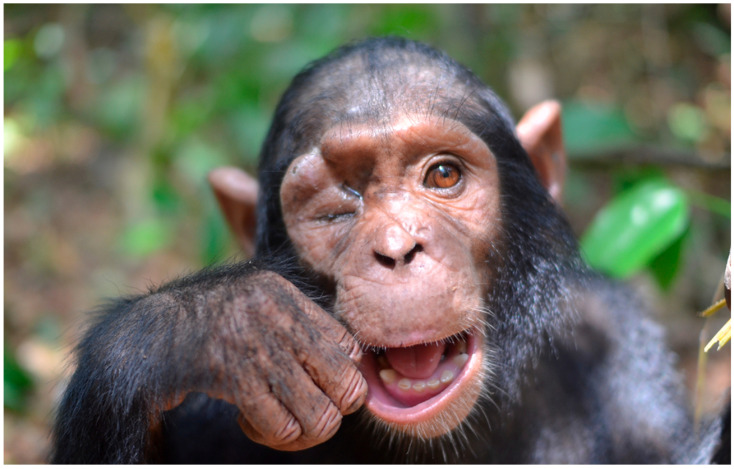
Initial clinical presentation of the infant chimpanzee, with right periocular swelling, suppurative discharge and a partially closed eyelid due to the formation of an orbital abscess.

A general examination of the chimpanzee was performed upon presentation to the wildlife rescue center. The chimpanzee presented with increased skin turgor, a dull coat, reduced appetite, lethargy, and malnutrition signs. Vital parameters including heart rate, respiratory rate and rectal temperature were within normal range [32,33]. There was a deep, linear laceration extending from the upper frontal region, across the supraorbital torus and frontal process, into the right orbital cavity. The right eyelid was permanently closed, with an open wound visible in the lateral area. Surrounding tissues were moderately inflamed, exhibiting localized heat, pain and suppuration (Figure 1).

Two days later, keepers observed right-sided periocular swelling accompanied by mucopurulent discharge. Initial treatment consisted of topical neomycin-polymyxin B-dexamethasone drops administered twice daily and systemic doxycycline (5 mg/kg twice daily for 10 days). Although the symptoms temporarily improved, they recurred. Repeat physical examinations under anesthesia revealed progressive exophthalmia, conjunctival edema, corneal opacity, and eyelid erythema.

For the performance of additional diagnostic procedures, including eye ultrasonography, blood work, electrocardiography, and urine collection, the animal was anesthetized with ketamine (3 mg/kg intramuscularly) and medetomidine (0.03 mg/kg intramuscularly). Ocular ultrasonography was performed using a high-frequency linear transducer. The procedure revealed a hypoechoic, fluid-filled right globe containing internal echogenic debris, accompanied by severe scleral thickening and disruption of the normal anatomical structures of the eye. These findings were consistent with chronic intraocular inflammation and abscess formation. No retrobulbar mass or foreign body was detected. The left eye appeared normal on ultrasound. Hematological analysis revealed mild neutrophilic leukocytosis (16 × 10^3^/L) and elevated globulin levels. Mild anemia was also detected, and the chimpanzee was typed as blood group O^+^, with hematocrit and erythrocyte count marginally below the reference interval for their species. All other hematological parameters, including mean corpuscular volume, lymphocyte, monocyte, eosinophil, and basophil counts, hemoglobin concentration and platelet count, were within the normal limits for *Pan troglodytes* [32]. Serum biochemistry revealed no abnormalities aside from elevated globulin concentration. Glucose, urea, creatinine, sodium, potassium, calcium, total protein, cholesterol, triglycerides, aspartate aminotransferase (AST), alanine aminotransferase (ALT), C-reactive protein, and serum ferritin values were all within the reference ranges for the species (Table 1) [32,33]. An electrocardiographic evaluation revealed a normal sinus rhythm with mild sinus tachycardia. This was considered a physiological response to stress and the pharmacological agents administered during the procedure. No arrhythmias, conduction abnormalities or repolarization disturbances were detected on ECG, and all waveform intervals and amplitudes fell within the reference limits for the species [34].

Urinalysis revealed a normal yellow-colored, clear sample, which was obtained by cystocentesis. No abnormalities were noted in terms of turbidity, or the presence of sediment. Dipstick analysis and microscopic examination of the sediment were unremarkable, indicating normal renal function and the absence of a urinary tract infection or hematuria. Parasitological analyses were carried out on both fecal and peripheral blood samples. Examination of the feces, including direct smear and flotation techniques, yielded no evidence of helminths, protozoa or oocysts. Blood smears were negative for hemoparasites such as *Trypanosoma* spp. or *Plasmodium* spp., and no microfilariae were observed.

Intravenous crystalloid fluids (Ringer’s lactate; 2.5 mL/kg/h; B. Braun Medical SAS, Boulogne, France) were administered continuously for the first 24 h to ensure adequate hydration and tissue perfusion. To prevent the intravenous catheter removal, the chimpanzee was continuously monitored, and protective bandaging was applied around the catheterisation site. Additionally, caretakers provided close supervision to ensure the catheter remained intact. Following stabilization, oral supplementation with a multivitamin complex containing vitamins A, B, E and essential minerals (Alpha-Vit^®^, East Asia Laboratories, Inc, San Mateo, Rizal, Filipinas, 5 mL, twice daily) was initiated, alongside oral rehydration salts (WHO formulation: 20 mEq/L sodium, 20 mEq/L potassium and 30 mEq/L chloride, 50 mL, twice daily) to support electrolyte balance and facilitate recovery. Antibiotic therapy with amoxicillin-clavulanic acid (Augmentin^®^, GlaxoSmithKline, London, UK) was started at a dose of 15 mg/kg PO TID for 14 days and metronidazole (Flagyl^®^, Sanofi, Paris, France) at a dose of 15 mg/kg PO BID for 14 days, to target potential pathogens associated with the infected ocular site. Wound care involved irrigating the periorbital area with sterile physiological saline, followed by application of topical gentamicin 0.1% ointment three times daily for 14 weeks. Nutritional support was provided through a high-calorie liquid diet, administered every two hours, consisting of mashed banana, human infant formula, cooked egg, cereal powder and diluted commercial fruit juice to ensure palatability and nutrient density. The animal received continuous nursing care and close behavioral monitoring by both veterinarians and caretakers.

The chimpanzee remained under observation at the rehabilitation center for twelve months following the initial presentation. During this period, three separate episodes of purulent discharge were noted emanating from the lateral canthus of the right eye, which was the site of the original lesion. The chimpanzee was treated with amoxicillin-clavulanic acid (Augmentin^®^, GlaxoSmithKline, Middlesex TW8 9GS, UK; 15 mg/kg orally three times a day for 14 days), ibuprofen (20 mg/kg orally twice a day for five days) and omeprazole (0.4 mg/kg orally twice a day for 14 days) as a gastric protectant on both occasions. The clinical signs resolved completely in both cases, with no residual inflammation or discomfort observed after each treatment course. Furthermore, the animal experienced three distinct episodes of upper respiratory tract infection at three-month intervals. These episodes were characterized by mild nasal discharge and a moderate, non-productive cough. They responded rapidly to symptomatic treatment with chlorpheniramine maleate (Surdex, IMEX Pharma, Abidjan, Ivory Coast; 50 mg/kg PO BID for five days), with complete remission achieved within 72 h in each case.

Following a third recurrence of purulent discharge, accompanied by localized heat, swelling, and evident discomfort in the right periorbital region, surgical intervention was considered necessary to confront the suspected underlying chronic infection. This occurred approximately 12 months after the initial presentation.

### 2.1. Anesthesia Technique

The animal was fasted for eight hours prior to the surgical procedure. General anesthesia was achieved using a dissociative protocol consisting of intramuscular ketamine (5 mg/kg; Kyron Laboratories, Johannesburg, South Africa) combined with medetomidine (0.05 mg/kg; Pfizer Animal Health, Kent, UK), following protocols commonly applied in field settings for chimpanzees [33]. Once adequate anesthesia was achieved, a cephalic vein catheter was placed to allow intravenous administration of lactated Ringer’s solution, delivered at a maintenance rate of 5 mL/kg/h throughout the procedure. Analgesia was provided with intramuscular tramadol (2 mg/kg) and a single intramuscular dose of diclofenac sodium (1 mg/kg) for its anti-inflammatory and analgesic properties. Diazepam (5 mg/kg IM) was administered to enhance muscle relaxation and provide additional anesthesia [33,35]. Local analgesia was achieved by instilling 1% lidocaine drops into the conjunctival sac of the affected eye prior to surgery. Anesthetic monitoring included continuous assessment of depth through jaw tone and ocular position, as well as continuously monitoring of heart rate, respiratory rate, non-invasive arterial blood pressure, electrocardiography, pulse oximetry and rectal body temperature. No physiological abnormalities or anesthetic complications were recorded during the procedure.

**Figure 2:**
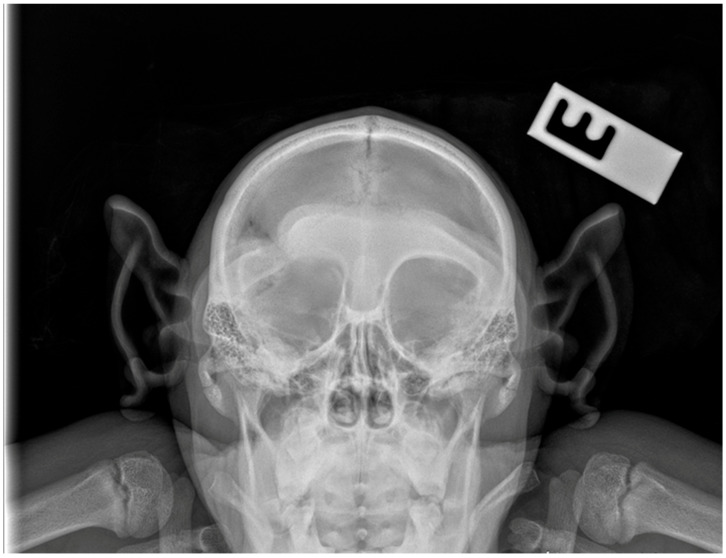
Dorsoventral skull radiograph showing increased soft tissue opacity in the right orbit and obliteration of the normal orbital fat planes. There is no evidence of bone lysis, periosteal reaction or foreign bodies.

Prior to the surgical procedure, a lateral and dorsoventral skull radiograph were performed under anesthesia to evaluate the extent and location of the lesion. Imaging revealed increased soft tissue opacity in the right periorbital region, accompanied by diffuse swelling and obliteration of the normal orbital fat planes, findings consistent with intraorbital abscess formation. There were no radiographic signs of osseous destruction, periosteal reaction, or foreign body presence. However, evaluation of the right supraorbital region demonstrated an area of irregular bone contour with evidence of callus formation, consistent with post-traumatic remodeling. This finding is compatible with a healed defect resulting from the original penetrating injury (Figure 2).

These findings, when integrated with the clinical presentation and history of recurrent episodes, supported the diagnosis of a chronic orbital abscess requiring definitive surgical management.

### 2.2. Surgical Technique

Once the chimpanzee was anesthetized, the surgical site was clipped and aseptically prepared using a 2% chlorhexidine scrub, applied in a concentric pattern to reduce the risk of contamination. The surgical procedure was performed using a transconjunctival enucleation approach [36,37,38,39] adapted to the orbital anatomy of the chimpanzee. After the periorbital region was prepared aseptically and draped, a lid speculum was gently applied. A 360-degree conjunctival peritomy was then performed at the limbus using tenotomy scissors. The conjunctiva, Tenon’s capsule and periorbital fascia were carefully dissected to expose the extraocular muscles. The rectus and oblique muscles were individually identified, isolated using fine hemostats and transected near their scleral insertions.

**Figure 3:**
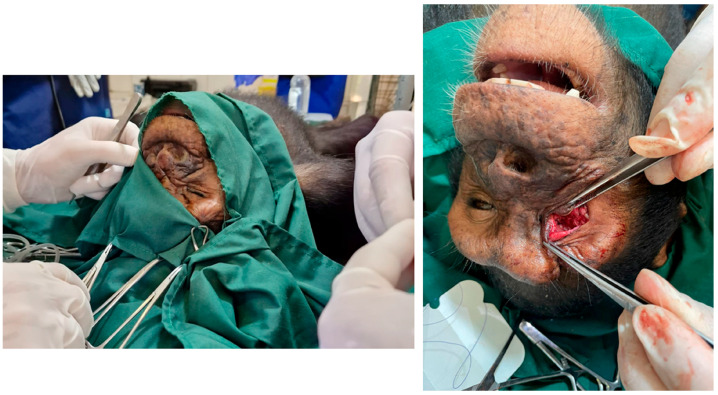
Intraoperative view of the enucleation procedure. The optic nerve and associated ophthalmic vessels are dissected at the orbital apex using fine surgical instruments.

**Figure 4:**
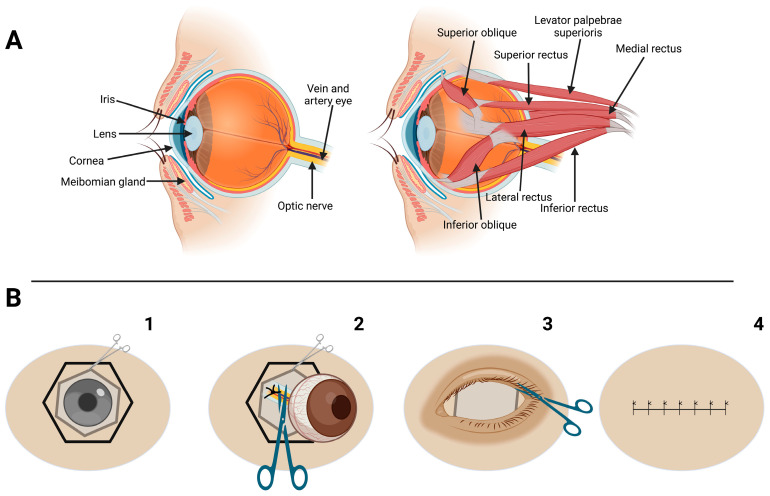
Ocular anatomy and key surgical steps during transconjunctival enucleation in a chimpanzee. (**A**) Anatomical representation of the eye and orbit: sagittal view (**left**) showing the course of the optic nerve and major vascular structures; and (**right**) illustrating the extraocular muscles. (**B**) Schematic representation of the main surgical steps in the transconjunctival enucleation procedure: B1, circumferential conjunctival peritomy at the limbus; B2, transection of the extraocular muscles and careful ligation of the neurovascular bundle (optic nerve and ophthalmic vessels) prior to transection at the orbital apex; B3, eyelid trimming; B4, surgical closure using an intradermal suture pattern.

Dissection was conducted with meticulous attention to preserving surrounding periorbital fat and avoiding trauma to vital neurovascular structures, including the ophthalmic branch of the trigeminal nerve and the internal ophthalmic artery. Hemostasis was maintained using Halsted hemostatic forceps throughout the procedure. The optic nerve, ophthalmic artery, and vein, located deep within the orbital apex, were gently elevated using a curved hemostat. These structures were then securely ligated using a 3-0 monofilament glyconate suture (Monosyn^®^, B Braun, Melsungen, Germany) and transected later on using curved Metzenbaum scissors. Extreme caution was exercised to prevent traction injury and hemorrhage, especially from the central retinal artery, which enters the globe via the optic nerve (Figure 3 and Figure 4).

After enucleation, the orbital cavity was carefully inspected for any residual purulent or necrotic material. A substantial quantity of soil and sand was discovered within the orbit. The area was therefore thoroughly rinsed with sterile isotonic saline to minimize the risk of postoperative infection. Closure was performed in layers to ensure structural integrity. The conjunctiva was sutured using a continuous pattern with 4-0 monofilament glyconate suture (Monosyn^®^, B. Braun). The palpebral margins were surgically trimmed to excise the superficial glandular tissue, including the Meibomian (tarsal) glands and the associated Zeis and Moll glands, in order to reduce the risk of postoperative cyst formation or secondary infection. The cutaneous closure was achieved using a continuous intradermal pattern with the same 4-0 monofilament suture, ensuring anatomical alignment and promoting optimal healing.

### 2.3. Postoperative Period

Postoperative management was designed to optimize recovery and minimize the risk of complications. The chimpanzee was housed in an isolated recovery enclosure adjacent to his social group, allowing for visual and auditory contact while preventing direct physical interaction. This arrangement helped to maintain environmental stability and minimize psychological stress, while also reducing the risk of accidental trauma or interference with the surgical site. Physical activity was restricted for a period of two weeks to ensure proper healing and to prevent suture disruption.

During the postoperative period, analgesic management included oral administration of tramadol (2 mg/kg twice daily) and a single intramuscular dose of diclofenac sodium (1 mg/kg) after anesthesia recovery, providing effective multimodal pain control. The surgical site was treated with topical antimicrobial therapy using gentamicin ointment applied twice daily. Systemic antibiotic coverage was also provided in the form of oral metronidazole (30 mg/kg PO BID for 10 days) and amoxicillin-clavulanic acid (15 mg/kg PO TID for 14 days), targeting both aerobic and anaerobic bacterial pathogens that can be involved in orbital infections.

**Figure 5:**
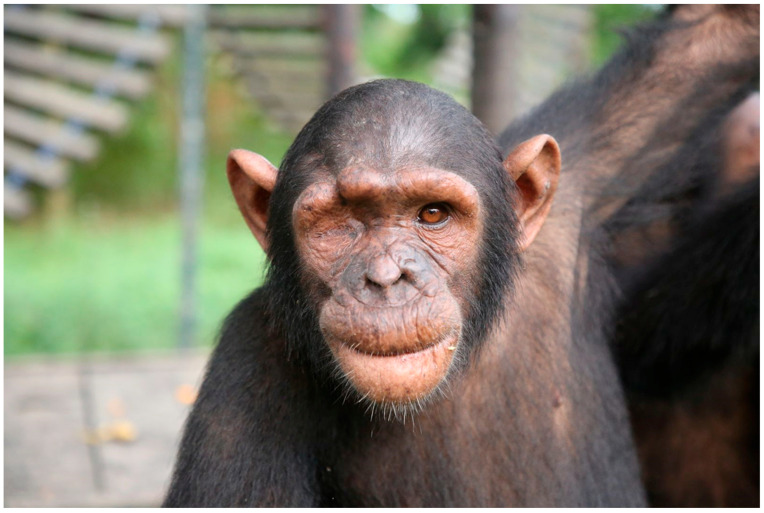
Postoperative appearance of the surgical site 2 months after enucleation, showing complete wound healing without signs of inflammation or infection.

To discourage self-injury and manipulation of the surgical wound, superficial sutures were strategically placed in non-critical areas, such as the extremities (e.g., legs and feet), serving as behavioral distractions. This approach, combined with continuous behavioral monitoring by the nursing team, reduced the risk of self-trauma or accidental suture disruption. In addition, the placement of these sutures was incorporated as part of an environmental enrichment strategy [40,41], further promoting the chimpanzee’s engagement and reducing focus on the surgical site. The animal made an uneventful full recovery. Normal feeding and social behaviors resumed within 48 h following the surgical procedure, indicating rapid postoperative adaptation. Functional recovery was assessed daily, with particular attention to wound appearance, general behavior, appetite, and mobility. At the 14-day follow-up examination, the surgical site had completely healed, showing no signs of inflammation, infection, or wound dehiscence. The tarsorrhaphy sutures remained intact, and no postoperative complications associated with the enucleation procedure were observed (Figure 5). At the 12-month follow-up, the chimpanzee showed full adaptation to monocular vision, maintaining normal feeding, locomotion, and social interactions without evidence of impaired welfare.

## 3. Discussion

This case report provides a comprehensive and novel description of the diagnostic approach, surgical procedure, and postoperative management of enucleation in a young chimpanzee. It represents one of the few detailed descriptions of this surgical intervention in great apes. Although ocular pathologies are underreported in non-human primates [13,18], this case highlights the importance of recognition and appropriate medical management of chronic periocular infections to prevent progression to orbital abscess formation and subsequent need for enucleation. Timely surgical intervention is crucial to alleviate discomfort and prevent further complications in great apes [29,32,42,43]. In this case, the procedure was performed approximately 12 months after the initial presentation, as the condition initially showed improvement but recurred, and following three such episodes, surgical intervention was deemed necessary.

Many chimpanzees arriving at wildlife sanctuaries exhibit pre-existing injuries and illnesses resulting from the illegal primate trade associated with the exotic pet market and bushmeat. These conditions commonly include fractures, deep lacerations, acute or chronic infections, and psychological trauma [6,7]. In the present case, it is highly likely that the mother was killed as part of bushmeat trafficking, and the infant sustained a machete wound during the violent encounter. In fact, the capture of a single infant chimpanzee may result in the death of five to ten adult chimpanzees [7].

Such cases present both ethical and practical challenges for veterinarians working in rescue centers, who are often required to make complex decisions regarding surgical interventions in contexts where resources are limited. Surgery on chimpanzees carries inherent anesthetic and surgical risks, which are exacerbated by the difficulties of postoperative care and monitoring in environments with limited medical infrastructure [4,6,7]. These procedures also require veterinarians with advanced surgical expertise and impose significant financial burdens on rehabilitation centers, where priorities must be carefully balanced between the welfare of individuals and broader conservation objectives [6,7]. In this case, however, the decision to proceed with surgery proved clinically justified. Although initial medical management temporarily alleviated symptoms, recurrent episodes of infection and purulent discharge were documented over a twelve-month period. Given the chronic nature of the condition, which may have been exacerbated by the presence of a deep, partially healed wound tract that communicated with the orbit, surgical intervention was therefore undertaken twelve months after the onset of clinical signs. When the tract was traumatized through self-grooming or environmental exposure, contaminants such as soil and debris were likely reintroduced into the wound bed, triggering recurrent infection and pain. The persistence of the infection despite prolonged antibiotic therapy, combined with evidence of discomfort and the risk of further complications, supported the decision to perform enucleation as definitive treatment, aiming to eliminate the source of the pain and minimize the risk of recurrence.

Performing enucleation on a chimpanzee involved unique challenges, as few detailed surgical guidelines exist for this species. The surgical team therefore relied heavily on the literature concerning human enucleation techniques [44] and comprehensive anatomical studies of the primate orbit [15,31]. Practical surgical experience from both small and exotic animal medicine was adapted to address the anatomical and behavioral specifics of chimpanzees within the constraints of a wildlife rescue center setting [30,31]. Unlike enucleations in small animals such as canine, where ligation of the optic nerve and ophthalmic vessels is typically unnecessary due to minimal hemorrhage encountered after hemostatic clamping [45,46], in chimpanzees and humans it is essential to ligate the optic nerve and the ophthalmic artery and vein [17,31,44]. This requirement arises from the larger caliber of these vessels, their close proximity within the confined orbital apex, and the robust vascular supply to the globe in great apes, all of which increase the risk of intraoperative hemorrhage if left unsecured [15,31]. Performing ligation prior to transection was crucial for achieving effective haemostasias and minimizing intraoperative blood loss, thereby enhancing surgical safety and procedural control in a resource-limited sanctuary environment.

Anesthetic management in chimpanzees is inherently complex, requiring species-specific protocols to ensure both safety and efficacy [27]. In this case, a combination of ketamine and medetomidine provided effective dissociative anesthesia, allowing for safe handling and surgical intervention. Perioperative analgesia was effectively managed using tramadol and diclofenac, ensuring adequate pain control and contributing to the animal’s comfort during the postoperative period. In addition to systemic analgesics, topical application of 1% lidocaine was instilled into the conjunctival sac prior to surgical manipulation. This provided effective local analgesia, complementing the systemic protocol and contributing to optimal intraoperative pain control. Continuous intraoperative monitoring of cardiovascular and respiratory parameters facilitated the early detection and management of potential complications, ensuring the animal’s stability throughout the procedure.

An important consideration during ocular surgery is the potential occurrence of the oculo-cardiac reflex, a trigeminovagal reflex that can lead to significant bradycardia and, in severe cases, cardiac arrhythmias [47,48]. Continuous intraoperative monitoring was therefore employed throughout the enucleation procedure. In this case, no bradycardic episodes or cardiac disturbances consistent with the oculo-cardiac reflex were observed. Although more frequently reported in human ophthalmic surgery, the reflex has also been described in animals, including non-human primates [27,28]. This highlights the importance of vigilant anesthetic monitoring and preparedness to manage potential vagal responses during ocular interventions in great apes.

Effective postoperative care for great apes requires constant monitoring to ensure adequate pain relief and prevent them from causing themselves trauma to the surgical site. To our knowledge, no peer-reviewed studies have formally evaluated the use of sutures in non-critical body areas to distract primates from their surgical wounds. However, this empirical strategy has been employed in sanctuaries and wildlife rescue settings, where Elizabethan collars are often impractical. In our case, it was incorporated as part of an environmental enrichment approach aimed at diverting attention from the primary surgical wound. Such techniques are widely recognized as essential for reducing stress, preventing self-trauma and promoting recovery in captive chimpanzees [40,41,49]. Close observation of feeding behavior, wound healing and social interactions was also essential in this case to ensure a successful outcome, highlighting the importance of dedicated nursing care and behavioral management during the postoperative period [42].

## 4. Conclusions

In conclusion, this case demonstrates that enucleation in chimpanzees can be successfully performed within sanctuary settings when medical management of ocular infections fails to resolve a chronic pathology. Careful case selection, meticulous surgical technique, tailoredanesthetic protocols and comprehensive postoperative management are critical to achieving a favorable outcome while ensuring animal welfare. This report contributes to the limited but growing body of veterinary literature on surgical interventions in great apes and underscores the importance of sharing detailed clinical experiences to inform and support future medical and surgical decision-making in wildlife sanctuary medicine.

## Figures and Tables

**Table 1:** Chimpanzee’s values and normal physiological, hematology, and biochemistry values in 2-year-old chimpanzees (*Pan troglodytes*).

Parameter	Values of the Chimpanzee	Normal Values (Young Chimpanzee ~2 Years)
Heart rate (bpm)	71	53–89
Respiratory rate (breaths/min)	27	25–40
Rectal temperature (°C)	37.4	37.0–38.5
Hematocrit (%)	42	36–46
Hemoglobin (g/dL)	12.8	11–14
Red blood cells (10^6^/µL)	5.2	4.5–6.0
White blood cells (10^3^/µL)	16	7–12
Platelets (10^3^/µL)	320	250–450
Urea (mg/dL)	11	5–15
Creatinine (mg/dL)	0.9	0.5–1.2
Glucose (mg/dL)	98	70–120
ALT (U/L)	34	20–50
AST (U/L)	46	25–60
Alkaline phosphatase (U/L)	280	150–400
Total proteins (g/dL)	7.2	6.0–8.0
Globulin (g/dL)	4.1	2.5–3.5
Albumin (g/dL)	4.0	3.0–4.5

Values based on data from Atencia, R. (2016) [32] and Atencia et al. (2017) [33].

## Data Availability

All data will be available upon request to the authors.

## Abbreviations

The following abbreviations are used in this manuscript:

PO	Oral route of medication administration
TID	Three times in a day
BID	Two times in a day
GOT	Aspartate aminotransferase
GPT	Alanine aminotransferase
